# Pre‐exposure prophylaxis in real life: experience from a prospective, observational and demonstration project among men who have sex with men in Benin, West Africa

**DOI:** 10.1002/jia2.26130

**Published:** 2023-06-12

**Authors:** Souleymane Diabaté, Luc Béhanzin, Fernand A. Guédou, Ella Goma‐Matsétsé, Marius Olodo, Marlène Aza‐Gnandji, Alban Dossouvo, Axel Akpaka, Elyote Chagas, Flore Gangbo, Djimon Marcel Zannou, Michel Alary

**Affiliations:** ^1^ Centre de recherche du CHU de Québec Université Laval Québec Québec Canada; ^2^ Département de médecine sociale et préventive Université Laval Québec Québec Canada; ^3^ UFR Sciences médicales Université Alassane Ouattara Bouaké Côte d'Ivoire; ^4^ École nationale de formation des techniciens supérieurs en santé publique et en surveillance épidémiologique Université de Parakou Parakou Benin; ^5^ Organisation pour la Promotion de la Santé et le Développement communautaire Cotonou Benin; ^6^ Dispensaire IST Centre de santé communal de Cotonou 1 Cotonou Benin; ^7^ Benin Synergie Plus Cotonou Benin; ^8^ Réseau Sida Bénin Cotonou Benin; ^9^ Programme Santé de Lutte contre le Sida Cotonou Benin; ^10^ Faculté des sciences de la santé Université d'Abomey‐Calavi Cotonou Benin; ^11^ Centre national hospitalier universitaire HMK Cotonou Benin; ^12^ Institut national de santé publique Québec Québec Canada

**Keywords:** Benin, HIV prevention, men who have sex with men, pre‐exposure prophylaxis, sub‐Saharan Africa, Truvada

## Abstract

**Introduction:**

Since many countries in sub‐Saharan Africa are willing to implement HIV oral pre‐exposure prophylaxis (PrEP) for men who have sex with men (MSM), data are needed to assess its feasibility and relevance in real life. The study objectives were to assess drug uptake, adherence, condom use and number of sexual partners, HIV incidence and trends in the prevalence of gonorrhoea and chlamydia.

**Methods:**

In this oral PrEP demonstration study conducted prospectively in Benin, a combination of tenofovir disoproxil fumarate‐TDF 300 mg and emtricitabine‐FTC 200 mg (TDF‐FTC) was offered daily or on‐demand to MSM. Participants were recruited from 24 August to 24 November 2020 and followed over 12 months. At enrolment, month‐6 and month‐12, participants answered to a face‐to‐face questionnaire, underwent a physical examination and provided blood samples for HIV, gonorrhoea and chlamydia.

**Results:**

Overall, 204 HIV‐negative men initiated PrEP. The majority of them (80%) started with daily PrEP. Retention rates at month‐3, 6, 9 and 12 were 96%, 88%, 86% and 85%, respectively. At month‐6 and month‐12, respectively, 49% and 51% of the men on daily PrEP achieved perfect adherence (self‐reported), that is seven pills taken during the last week. For event‐driven PrEP, the corresponding proportions for perfect adherence (last seven at‐risk sexual episodes covered) were 81% and 80%, respectively. The mean number (standard deviation) of male sexual partners over the last 6 months was 2.1 (1.70) at baseline and 1.5 (1.27) at month‐12 (*p*‐value for trend <0.001). Consistent condom use during the last 6 months was 34% (enrolment), 37% (month‐6) and 36% (month‐12). Three HIV seroconversions (2‐daily and 1‐event‐driven) were recorded. Crude HIV incidence (95% confidence interval) was 1.53 (0.31−4.50)/100 person‐years. *Neisseria gonorrhoeae* and/or *Chlamydia trachomatis* prevalence at the anal and/or pharyngeal and/or urethral sites was 28% at baseline and 18% at month‐12 (*p*‐value = 0.017).

**Conclusions:**

In West Africa, oral PrEP introduction in routine practice as a component of a holistic HIV prevention package is feasible and may not result in a significant increase in condomless sex among MSM. Since HIV incidence was still higher, additional interventions, such as culturally tailored adherence counselling, may be needed to optimize the benefits of PrEP.

## 1 INTRODUCTION

To curtail the human immunodeficiency virus (HIV) epidemic across the world, tremendous efforts are being invested in various integrated preventive and treatment interventions. During recent decades, 117 million new infections and 16.6 million AIDS‐related deaths have been averted worldwide due to condom use and antiretroviral therapy [1]. People who inject drugs, female sex workers (FSW), transgender women, gay men and other men who have sex with men (MSM) and their sexual partners are still disproportionately affected by the HIV epidemic, due to a combination of factors, such as stigma, discrimination and criminalization [1]. In 2021, 70% of the 1.5 million new HIV infections estimated worldwide occurred among these vulnerable populations. During the same year, the risk of acquiring HIV was 28 times greater among gay men and other MSM than among heterosexual men [2].

In 2015, to reduce the HIV burden, the World Health Organization (WHO) recommended extending oral pre‐exposure prophylaxis (PrEP) containing tenofivir disoproxil fumarate (TDF) to people at substantial risk of HIV, including MSM [3]. PrEP has proven to be an effective HIV prevention method, especially when adherence is good [4, 5]. However, access to PrEP remains minimal in some regions of the world, such as sub‐Saharan Africa [1, 4, 6]. Poor access to PrEP in this region may conceal contextual issues that need to be assessed before scaling up PrEP. Also, PrEP could lead to lower condom use or risk compensation and to sub‐optimal adherence in real‐life conditions [7, 8, 9]. Hence, we initiated a series of studies in Benin, West Africa, to provide information about oral PrEP adoption by national health authorities. In this country, HIV prevalence is higher among MSM (7.0%) and FSW (8.5%) than in the general adult population (0.8%) [1]. In a demonstration study on daily oral PrEP that we conducted among FSW, from October 2014 to December 2018, optimal adherence was low: 56% based on self‐reports and 27% for plasma drug concentrations [10, 11]. Concerning MSM, the first study that we designed to assess the intention to use PrEP and its barriers and facilitators showed that the majority of Beninese MSM were willing to adopt oral PrEP if made available [12]; and that free access within MSM networks was an important facilitator, while a decrease in condom use was not seen as a hindrance [13].

In light of these results, we initiated this demonstration study of oral PrEP (a generic combination of tenofovir disoproxil fumarate‐TDF 300 mg/emtricitabine‐FTC 200 mg; TDF‐FTC) among Beninese MSM. Oral PrEP was added to an HIV prevention package, comprised of clinical and behavioural components, already offered to MSM in Benin. The objectives of the study were to assess uptake, retention and adherence to PrEP, trends in consistent condom use and number of sexual partners, as well as HIV incidence and trends in the prevalence of gonorrhoea and chlamydia during follow‐up.

## 2 METHODS

### 2.1 Study design, setting and population

This oral PrEP demonstration project was an observational cohort study conducted prospectively in Cotonou and its suburbs. The MSM of Cotonou, the largest city of Benin, are organized into two main networks, that are *Réseau Bénin Synergie Plus* (BeSyP) and *Réseau Sida Bénin* (RSB). An investigation carried out in 2017 by BeSyP reported 3391 MSM in the study catchment area [14]. Since HIV prevalence among MSM is estimated at 7.0% [15], there should be around 3000 HIV‐negative MSM in the study area. The study centre, *Dispensaire IST*, was the first public clinic in Benin dedicated to people at high risk of acquiring HIV. For many years, it has been offering an integrated HIV control package to FSW, MSM and their sexual partners. The package consists of condom distribution, non‐governmental organizations’ awareness campaigns within the community, sexually transmitted infections (STI) screening/treatment, as well as early antiretroviral therapy initiation at the clinic. Ordinarily, MSM are recommended to visit the clinic monthly and whenever they have any health issues. The study employed a community‐participatory approach with an advisory platform composed of 10 representatives from the study team and seven civil society or governmental organizations.

A community mobilization phase, led by 10 peer educators from BeSyP and RSB, preceded enrolment in the study. This team included two supervisors, one from each organization. The sensitization messages were about the study objectives and procedures; the effectiveness, options and adherence to PrEP; HIV self‐testing and linkage to the clinic; and other STI screening and treatment. All these activities were provided free of charge. The 10 men received training on how to monitor peers’ sexual activity and risk‐taking on a monthly basis, manage PrEP side effects, fill the study tools and make referrals to the study centre whenever needed, such as when a participant had STI symptoms. During the mobilization phase in the community, every MSM reporting himself as being HIV negative was given a voucher with a serial number and the phone number of a supervisor. Each supervisor was in charge of the men affiliated with his organization. For each HIV‐negative man who agreed to take a voucher in the field, the supervisors organized an appointment at *Dispensaire IST*. Men unable to indicate their HIV status did not receive a voucher.

Men eligible to participate in the study were those born male, aged ≥18 years, members of BeSyP or RSB (who identified themselves as MSM) and HIV negative. Since generic TDF‐FTC is ineffective against hepatitis C virus (HCV), people with chronic hepatitis C (anti‐HCV antibodies—IgG) were not included in the study and were referred to the main University Hospital Centre of Cotonou for follow‐up according to national guidelines. Men with a creatinine clearance <60 ml/minute (Cockcroft−Gault equation) were also excluded. The sample size, estimated at 200 men for financial reasons, was large enough to assess, for any outcome of interest, a prevalence of 50% (conservative value), with a precision of 7%. Participants were recruited through a two‐stage sampling procedure. First, 7/13 districts were picked randomly with a probability proportional to size. Second, a fixed number of eligible men were selected consecutively from each, using a random route sampling procedure [16]. In short, from the study centre, a direction was selected randomly and, based on the mapping carried out by BeSyP in 2017 and the updated list of networks’ members, all households in the selected direction with an MSM were visited by the peer educators. To account for non‐eligibility, the number of participants reached in the field was extended to 266 (33% increase from 200).

### 2.2 Enrolment and follow‐up procedures

Recruitment took place at the clinic between 24 August and 24 November 2020. All MSM who attended the *Dispensaire IST* with a referral voucher were tested for HIV and evaluated for participation. HIV retesting was based on One Step Multi‐Infectious Disease Test (HBsAg/HCV/HIV/TP; InTec Products, Inc., Xinyang, China) followed by SD Bioline. Enrolled MSM had to be HIV negative for both InTec and SD Bioline tests. At the outset of the study, participants underwent a physical examination and provided rectal and pharyngeal swabs, as well as urine samples to test for *Neisseria gonorrhoeae* (NG) and *Chlamydia trachomatis* (CT) using GeneXpert (CEPHEID) (Table S1). Hepatitis B virus (HBV), HCV and syphilis were assessed using blood samples: rapid immuno‐chromatographic test for HBV surface antigen (HBsAg) and HCV antibodies; enzyme immunoassays (EIA) for HBV core (anti‐HBc) and surface (anti‐HBs) antibodies; and a rapid treponemal test followed by a rapid plasma reagin test if positive (syphilis).

The study was extended over 12 months and participants were followed every 3 months (Table S1). Men wishing to quit before month‐12 underwent the final study procedures during their last visit at the clinic. The last patient was seen in December 2021. Scheduled visits at month‐3 and month‐9 were conducted in the field (home, event‐venue, office of BeSyP or RSB) by peer educators. The main laboratory test for these two visits was HIV self‐testing with a referral to the clinic if positive. Participants received training on self‐testing and different explanation tools in French, English and Fon, the main local language. The tests were distributed free of charge. Normally, HIV self‐testing was performed at home by the participants. Upon request, a peer educator would assist them to perform the test, either at home or at a mutually agreed upon location. At month‐6 and month‐12, participants returned to the *Dispensaire IST* for a clinical examination and NG/CT/HIV testing as done at the study entry. To ensure that a man with an HIV‐positive test during follow‐up was not already infected at study entry, a third‐generation EIA antibody test, a Western Blot and a viral load measure were carried out on a blood sample collected at study entry and stored at the clinic. At enrolment, month‐6 and month‐12, a standard questionnaire on sexual behaviour, risk‐taking and adherence to PrEP was administered at the clinic by trained investigators. Additional unscheduled visits at the clinic were allowed at any time. Field visits were conducted by peer educators to seek out men who did not attend a follow‐up visit.

### 2.3 TDF‐FTC options and outcomes

Participants were free to choose the PrEP regimen they preferred. Daily intake consisted of taking one pill every day, while event‐driven PrEP consisted of four pills (2+1+1): two pills 2–24 hours before sex, one after 24 hours and the fourth after 48 hours. Men with active HBV were all recommended daily PrEP and, at the end of the study, they were referred to the main University Hospital Centre of Cotonou in order to continue the treatment as per national guidelines. Participants were asked to report all switches between the methods to the study staff. At study entry, irrespective of the type of intake, each man received a 30‐pill bottle of TDF‐FTC. Refills were done monthly for daily PrEP. For the event‐driven method, the participant's risk and ability to adhere to treatment were evaluated jointly with the study physician. Afterwards, the physician developed a follow‐up sheet indicating when to refill the bottle of TDF‐FTC. The stocks of TDF‐FTC were stored at the *Dispensaire IST* under the supervision of a trained nurse. At enrolment, she directly provided the participants with a bottle of TDF‐FTC. During follow‐up and based on the refill sheets created by the physician, the peer educators would pick up the pills from the nurse and provide them to the participants in the field. Each month, all men received adherence support, condoms and lubricant gel. These services were free of charge. At most, each peer educator was responsible for 20 MSM. The sensitization messages delivered by the peer educators during the mobilization phase and the HIV preventive and treatment package offered at the *Dispensaire IST* were maintained during follow‐up.

The main outcomes were: uptake, the proportion of recruited MSM who started daily or event‐driven PrEP; retention rates, the proportion of men attending each scheduled visit at the clinic; self‐reported adherence at months 6 and 12, defined as perfect (proportion of men who reported seven pills during the last week for daily PrEP and all last seven at‐risk sexual episodes covered for event‐driven PrEP), or partial (at least four pills taken during the last week for daily PrEP and 3/7 last at‐risk sexual episodes covered for event‐driven PrEP); the proportion of men who reported consistent condom use over the last 6 months; the number of sexual partners reported during the last 6 months; HIV incidence, seroconversion was postulated to occur at the mid‐point between the follow‐up visit during which the test was positive and the previous visit where it was negative; and, finally, the prevalence of anal, urethral and oral gonorrhoea and chlamydia, which were assessed at each scheduled visit at the clinic.

### 2.4 Statistical analysis

The data were checked for completeness and validity before data entry with the EpiData software using a double‐entry system. Participants’ characteristics were described using means (±standard deviation) and proportions for continuous and categorical factors, respectively. To account for dependency during follow‐up, paired *t*‐tests and the Mc Nemar chi‐square tests were used when indicated, and time trends for variables, such as adherence and risk‐taking, were investigated using generalized estimating equations with an unstructured matrix. A Poisson approximation method enabled the assessment of HIV incidence (95% confidence interval, 95% CI). The statistical analyses were done with SAS version 9.4 (SAS Institute Inc, Cary, NC, USA).

### 2.5 Ethical considerations

The ethics committee of the CHU de Québec–Université Laval in Québec, Canada, and the Benin National Ethics Committee for Health Research approved the study protocol. Participation was subject to written informed consent, and withdrawal was possible. Participants received about 8 USD at each visit to compensate for their travel cost and time devoted to the study procedures. They received, free of charge, a comprehensive package of friendly services targeting STI. Unvaccinated participants without an active or a lifetime hepatitis B infection were offered free vaccination.

## 3 RESULTS

### 3.1 Baseline characteristics

**Figure 1:**
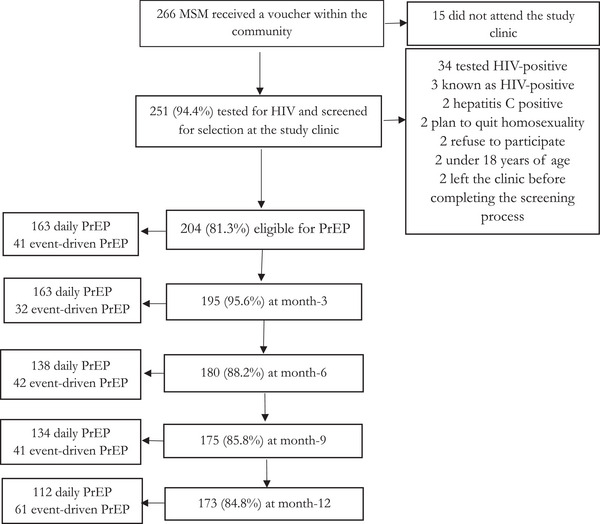
Flow chart for selection and follow‐up in a PrEP demonstration project conducted among MSM, Cotonou, Benin, 2020–2021.

Of the 266 MSM who received a voucher, 15 did not attend the clinic (Figure 1). Hence, 251 men (95%) were evaluated for enrolment and 47 were excluded for various reasons, such as testing HIV positive (34 men). Finally, 204 HIV‐negative men initiated PrEP. Beninese men represented 96% of the study sample. The proportion of participants living as a couple with a woman was 13% (Table 1). The mean age (±sd) was 25.9 (±4.76) years. Overall, 7.3% of the participants did not attend secondary school. One hundred and sixteen men (57%) and 67% of all participants reported being bisexual and having at least two concurrent sexual partners at recruitment, respectively. One man out of five reported receptive sex as the main sexual role. One hundred and sixty‐three participants (80%) started with daily PrEP.

**Table 1:** Baseline characteristics of 204 MSM participating in an oral PrEP demonstration project, Cotonou, Benin, 2020–2021

Characteristics	Total ; *N* = 204 *n* (%)	Uptake of daily PrEP; *N* = 163 *n* (%)	Uptake of event‐driven PrEP; *N* = 41 *n* (%)
**Socio‐demographic**			
**Age in years**			
18−24	89 (43.6)	69 (42.3)	20 (48.8)
25−29	70 (34.3)	54 (33.1)	16 (39.0)
≥30	45 (22.1)	40 (24.6)	5 (12.2)
**Nationality**			
Benin	195 (95.6)	156 (95.7)	39 (95.1)
Other	9 (4.4)	7 (4.3)	2 (4.9)
**Religion**			
Catholic	167 (81.9)	130 (79.8)	37 (90.3)
Islam	26 (12.7)	23 (14.1)	3 (7.3)
Other	11 (5.4)	10 (6.1)	1 (2.4)
**Marital status**			
Married	19 (9.3)	18 (11.1)	1 (2.4)
Single	165 (80.9)	133 (81.6)	32 (78.1)
Cohabiting	15 (7.4)	10 (6.1)	5 (12.2)
Other	5 (2.4)	2 (1.2)	3 (7.3)
**Co‐resident**			
None (living alone)	51 (25.0)	39 (23.9)	12 (29.3)
Family member	115 (56.4)	96 (58.9)	19 (46.3)
Spouse/female sex partner	17 (8.3)	15 (9.2)	2 (4.9)
Male sex partner	7 (3.4)	5 (3.1)	2 (4.9)
Other	14 (6.9)	8 (4.9)	6 (14.6)
**Living as a couple**			
No	137 (67.2)	112 (68.7)	25 (61.0)
Yes, with a man	40 (19.6)	27 (16.6)	13 (31.7)
Yes, with a woman	16 (7.8)	16 (9.8)	0 (0.0)
Yes, with a woman and a man	8 (3.9)	5 (3.1)	3 (7.3)
No answer	3 (1.5)	3 (1.8)	0 (0.0)
**Education**			
None/primary	15 (7.4)	12 (7.4)	3 (7.3)
Secondary	91 (44.6)	74 (45.4)	17 (41.5)
Postsecondary	98 (48.0)	77 (47.2)	21 (51.2)
**Occupation**			
None	14 (6.9)	11 (6.8)	3 (7.3)
Student	53 (26.0)	40 (24.5)	13 (31.7)
Civil servant	31 (15.2)	26 (15.9)	5 (12.2)
Other	106 (51.9)	86 (52.8)	20 (48.8)
**Monthly revenue (FCFA)**			
<40,000	89 (43.6)	70 (42.9)	19 (46.3)
40,000−50,000	34 (16.7)	29 (17.8)	5 (12.2)
50,001−80,000	33 (16.2)	27 (16.6)	6 (14.6)
80,001−120,000	30 (14.7)	21 (12.9)	9 (22.0)
> 120,000	17 (8.3)	15 (9.2)	2 (4.9)
No answer	1 (0.5)	1 (0.6)	0 (0.0)
**Sexual behaviour**			
**Sexual preference**			
Homosexual	76 (37.2)	62 (38.0)	14 (34.1)
Bisexual	116 (56.9)	94 (57.7)	22 (53.7)
Heterosexual	11 (5.4)	7 (4.3)	4 (9.8)
No answer	1 (0.5)	0 (0.0)	1 (2.4)
**Having more than one sexual partner (sexual concurrency) at baseline**			
Yes	136 (66.7)	105 (64.4)	31 (75.6)
No	68 (33.3)	58 (35.6)	1 (24.4)
**Sexual role**			
Active or insertive	129 (63.2)	103 (63.2)	26 (63.4)
Passive or receptive	41 (20.1)	33 (20.2)	8 (19.5)
Versatile (one or another)	34 (16.7)	27 (16.6)	7 (17.1)
**Sexual role procuring more pleasure**			
Active or insertive	131 (70.1)	102 (68.5)	29 (76.3)
Passive or receptive	52 (27.8)	44 (29.5)	8 (21.1)
Versatile (one or another)	4 (2.1)	3 (2.0)	1 (2.6)
**Active HBV (HBsAg positive)**	18 (8.8)		
**Lifetime HBV (Ag HBs or anti‐HBc positive)**	77 (37.7)		
**Active syphilis^a^**	0		

Abbreviations: Ag, antigen; Anti‐HBc, antibody to hepatitis B core antigen; FCFA, *Franc de la Communauté Financière Africaine* (1 US dollar~500 FCFA, local currency); HBV, hepatitis B virus; MSM, men who have sex with men; *n*/*N*, number; PrEP, pre‐exposure prophylaxis.

^a^ There was one participant positive for the rapid treponemal test but not for the rapid plasma reagin one.

Active HBV (HBsAg‐positive) and lifetime HBV (HBsAg or anti‐HBc‐positive) were diagnosed among 8.8% and 38% of study participants, respectively. Only one man had a positive serum sample for the rapid treponemal test, but not for the rapid plasma reagin one.

### 3.2 Retention, adherence and adverse events

The retention rates at month‐3, 6, 9 and 12 were 96%, 88%, 86% and 85%, respectively (Figure 1). Sixty‐two participants switched from daily to event‐driven PrEP or vice‐versa, at least once. The mean number (±sd) of switches was 1.7 (±1.01). After enrolment, five men moved abroad or to cities far from Cotonou and did not complete any follow‐up visit.

**Figure 2:**
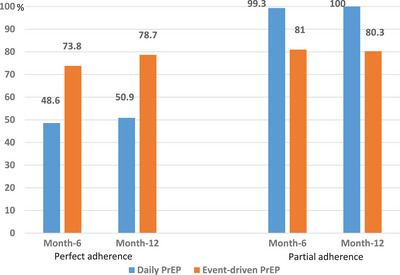
Self‐reported oral PrEP adherence among 204 MSM participating in a demonstration project, Cotonou, Benin, 2020–2021.

At month‐6 and month‐12, approximately, one man out of two taking daily PrEP and three out of four taking event‐driven PrEP achieved perfect adherence (Figure 2). There was no major difference over time. When considering a lower threshold for adherence, 90% of men using daily PrEP took at least four pills during the last week, while only 20% of participants in the event‐driven group failed to cover more than three out of the last seven at‐risk sexual episodes.

No death or serious clinical adverse event was reported. Overall, 70% of men at month‐6 and 53% at month‐12 reported minor adverse events, such as polyphagia, fatigue and muscle weakness.

### 3.3 Sexual behaviour and sexually transmitted infections

The number of male sexual partners reported over the last 6 months decreased significantly (Table 2). The mean number (±sd) was 2.1 (±1.70) at month‐0, 1.8 (±1.55) at month‐6 and 1.5 (±1.27) at month‐12 (*p*‐value for trend <0.001). At baseline and at each follow‐up visit, about two‐thirds of the participants reported inconsistent condom use.

**Table 2:** Number of sexual partners and condom use as reported by 204 MSM participating in an oral PrEP demonstration project, Cotonou, Benin, 2020–2021

	Study entry, *N* = 204	Month‐6, *N* = 180	Month‐12, *N* = 173	
**Number of male sexual partners, last 6 months**	***n* (%)**	***n* (%)**	***n* (%)**	***p*‐value**
0	17 (8.3)	20 (11.1)	21 (12.1)	<0.001
1	78 (38.2)	91 (50.5)	83 (48.0)	
2–4	93 (45.6)	57 (31.7)	64 (37.0)	
≥5	16 (7.9)	12 (6.7)	5 (2. 9)	
**Condom use during anal sex, last 6 months**				
Always	70 (34.3)	67 (37.2)	62 (35.8)	0.882
Not always	134 (65.7)	113 (62.8)	111 (64.2)	

Abbreviations: *N*, number; %, proportion.

Three HIV seroconversions (2‐daily and 1‐event‐driven) occurred during follow‐up (195.65 person‐years). A fourth seroconversion in the daily PrEP group was actually a prevalent case, according to the result of the viral load test. The test was done on the blood sample that was collected at baseline and stored. The EIA and Western blot tests on baseline samples were both negative for this participant. Crude HIV incidence (95% CI) was 1.53 (0.31–4.50)/100 person‐years. NG and/or CT prevalence, at one or more anatomic sites, was 28% at baseline and 18% at month‐12 (Table 3, *p*‐value for trend = 0.017). Over the same period, *p*‐values for the trend for NG alone and CT alone at one or more sites were 0.395 and 0.006, respectively. Overall, 8.8% of men with NG and/or CT infections were symptomatic.

**Table 3:** Prevalence of sexually transmitted infections among HIV‐negative MSM participating in a PrEP demonstration study, Benin, 2020–2022

	Month‐0 (*N* = 204); *n* (%)				Month‐6 (*N* = 180); *n* (%)				Month‐12 (*N* = 173); *n* (%)			
	Anal	Oral	Urethral	At least, one site	Anal	Oral	Urethral	At least, one site	Anal	Oral	Urethral	At least, one site
**NG**	20 (9.8)	13 (6.4)	5 (2.5)	31 (15.2)	8 (4.4)	4 (2.2)	5 (2.8)	13 (7.2)	13 (7.5)	9 (5.2)	5 (2.9)	22 (12.7)
**CT**	18 (8.8)	5 (2.5)	20 (9.8)	38 (18.6)	13 (7.2)	1 (0.6)	12 (6.7)	25 (13.9)	11 (6.4)	2 (1.2)	5 (2.9)	15 (8.7)
**NG and/or CT**	32 (15.7)	17 (8.3)	22 (10.8)	57 (27.9)	18 (10.0)	5 (2.8)	16 (8.9)	34 (18.9)	20 (11.6)	11 (6.4)	9 (5.2)	31 (17.9)

Note: For NG, CT and their combination (NG and/or CT): *p*‐values comparing month‐0 to month‐6 were NG (0.014), CT (0.211), NG and/or CT (0.037; *p*‐values comparing month‐6 to month‐12 were NG (0.084), CT (0.122), NG and/or CT (0.8147); and *p*‐values for trend were NG (0.395), CT (0.006), NG and/or CT (0.017).

Abbreviations: CT, *Chlamydia trachomatis*; NG, *Neisseria gonorrhoeae*.

## 4 DISCUSSION

In this demonstration study, 80% of men chose the daily PrEP option at baseline and almost 85% of all participants were seen at month‐12. In another oral‐PrEP demonstration study conducted in four community‐based clinics in West Africa (Abidjan‐Côte d'Ivoire, Bamako‐Mali, Lomé‐Togo and Ouagadougou‐Burkina Faso), 74% of MSM chose event‐driven PrEP [17]. Men recruited in our study were members of the two main MSM networks in Cotonou. As compared to other MSM, they might be easier to reach with the integrated HIV preventive package already offered in the study catchment area. Accordingly, they could be more aware of oral PrEP and inclined to use it on a daily basis [18]. Beninese MSM have already described the availability of PrEP within identity networks as an important facilitator for its use [13]. In some West African countries, such as Benin, Burkina Faso and Côte d'Ivoire, where specific laws penalizing same‐sex relations in private have been decriminalized or never existed [2], but where MSM face stigmatization and discrimination, identifying networks may play a core role in the successful implementation of PrEP by raising awareness among their members while helping them to stand up for their rights. In line with previous findings, MSM of Cotonou may contribute to “bisexual bridges” for the spread of STI since several participants were living with a woman or reported bisexuality as their sexual preference [19]. As reported elsewhere [17], some men switched from daily to event‐driven PrEP and vice‐versa. This suggests that MSM are continually adjusting the drug regimen to their daily living conditions and risk‐taking [17, 20]. As such, continuous assessment of oral PrEP retention rates in medical clinics may be of limited interest [21, 22]. In different contexts where low retention rates have been reported, there is no evidence that HIV incidence has increased significantly due to PrEP [17, 23].

About 50% of men in the daily PrEP group and 20% in the event‐driven one did not achieve perfect adherence, based on self‐reports. Perfect and partial adherence to event‐driven PrEP were higher and comparable. Concerns about being considered by sexual partners, friends or parents as HIV positive (taking treatment every day could be associated with HIV) or not being sexually active during a period of time may have contributed to lower adherence levels in the daily PrEP group [5, 24]. When considering all self‐reported adherence measures in the multi‐country study conducted in Abidjan, Bamako, Lomé and Ouagadougou, optimal adherence to event‐driven and daily PrEP was 41% and 71%, respectively [17]. The affiliation with MSM networks could explain why adherence was generally better in Cotonou than in the four cities. In spite of acceptable retention rates, the delivery of TDF‐FTC within the community and continuing counselling, adherence to PrEP remained low. Theoretical behavioural models addressing modifiable factors of adherence are requisite to the development of multi‐component and culturally sensitive interventions to enhance adherence [25]. Adherence is a more important issue for PrEP effectiveness than retention in medical clinics [26, 27, 28].

The number of male sexual partners reported over the last 6 months decreased from baseline to month‐12 (*p*‐value <0.001). In addition, consistent condom use as reported by men did not decrease. These results are in line with the prevalence of NG/CT that was high at enrolment and decreased during follow‐up. The decrease was mainly due to CT. Accordingly, one can assert that HIV risk‐taking did not worsen as observed elsewhere [29]. The absence of risk compensation may be due to the community‐based awareness interventions implemented prior to and during the study [17]. During clinical examination at enrolment and follow‐up visits, men with clinical signs of gonorrhoea and chlamydia were treated. This could have contributed to a decrease in these infections’ prevalence during follow‐up. HIV incidence was similar to that of the study conducted in Abidjan, Bamako, Lomé and Ouagadougou [17]. Also, as in those four cities, one prevalent case initiated daily PrEP. Even though this may concern very few people in a large‐scale PrEP programme, it adds to the debate on the reinforcement of screening strategies for people initiating PrEP in order to avoid drug resistance [30].

This community‐based study of a real‐world oral PrEP experience among MSM has limitations. After completing the enrolment process, five HIV‐negative men left Cotonou and did not do any follow‐up visit. HIV incidence could have been underestimated if at least one of them had seroconverted. Participants may have underreported receptive anal sex because it is associated with stigma [31]. Social desirability could have led to the high levels of self‐reported adherence and, especially, to the minimal difference between perfect and partial adherence to event‐driven PrEP [10]. However, it could be expensive to use more reliable tools, such as laboratory testing, to measure adherence in routine practice. The linkage of the study participants to identity networks should be taken into account. Men affiliated with such networks are probably easier to reach. Accordingly, they could have better knowledge of PrEP as compared to other MSM [32]. For this reason, the generalization of the findings to all MSM living in Benin could be limited. For instance, the availability of PrEP within MSM networks was significantly associated with its acceptability in the study setting [13]. During follow‐up, participants were not tested for new hepatitis C infections and we did not perform HBV viral load. Finally, retention rates were not assessed according to the PrEP regimen.

## 5 CONCLUSIONS

In West Africa, oral PrEP implemented in routine practice as a component of a holistic HIV prevention package is feasible and may not result in a significant increase in condomless sex among MSM. Since HIV incidence was still higher, additional interventions, such as culturally tailored adherence counselling, may be needed to optimize the benefits of PrEP.

## COMPETING INTERESTS

TDF‐FTC was a generic drug, Macleods Pharmaceuticals Ltd, bought in Benin at the Centrale d'Achat des Médicaments Essentiels et consommables médicaux (CAME). Both institutions as well as the funding ones, the Canadian Institutes of Health Research and PLAN International Bénin, were not involved in the study design, the analysis and interpretation of the data. Hence, the views expressed here are those of the authors and they are not necessarily in line with the official positions or policies of these institutions.

## AUTHORS’ CONTRIBUTIONS

MA, SD, LB, FAG, FG and DMZ conceived the study. AD contributed to the supervision of all activities in the field and at the study clinic. AA and EG contributed to all community activities. EG‐M contributed to the lab tests. MO, MA‐G, SD, MA and LB contributed to data analyses. The first version of the manuscript was written by SD and revised by MA. All authors read and approved the final version of the manuscript.

## FUNDING

This study was funded by the Canadian Institutes of Health Research (CIHR; grant # FDN‐143218) and Plan International Benin (Contracts #2291/PIB/CO/PO‐BJ06627 and #2508/Plan Int'l. BEN/CO/PO‐BJ06716).

## Supporting information

Additional files
**Table S1**. Study procedures applied to 204 MSM participating in an oral PrEP demonstration project, Cotonou, Benin, 2020–2021

## Data Availability

The data will be available on request from the authors if the manuscript is accepted for publication.
