# Supplementary material for: Pre‐exposure prophylaxis in real life: experience from a prospective, observational and demonstration project among men who have sex with men in Benin, West Africa
Source: J Int AIDS Soc. 2023 Jun 12;26(6):e26130. doi: 10.1002/jia2.26130 (PMC10258860; doi:10.1002/jia2.26130)
Supplement: Supplementary file 1 — Additional files Table S1. Study procedures applied to 204 MSM participating in an oral PrEP demonstration project, Cotonou, Benin, 2020–2021 [file JIA2-26-e26130-s001.docx]

**Table S1**. Study procedures applied to 204 MSM participating in an oral PrEP demonstration project, Cotonou, Benin, 2020-2021

| **Procedure / Visit** | **Enrollment** | **Month-3** | **Month-6** | **Month-9** | **Month-12** |
| --- | --- | --- | --- | --- | --- |
| Written informed consent | x |  |  |  |  |
| Questionnaire ^†^ | x | x | x | x | x |
| HIV testing ^‡^ | x | x | x | x | x |
| Clinical examination with syndromic diagnosis of STI | x |  | x |  | x |
| Anal and pharyngeal swabs and urine sample for NG/CT screening using GeneXpert (Cepheid) | x |  | x |  | x |
| Blood sample for creatinine | x |  | x |  | x |
| Blood sample (fingerpick and venous blood) for HBV/HCV ^§^ | x |  |  |  |  |
| Serum sample for Syphilis using two consecutive tests : Rapid Plasma Reagin test if the rapid treponemal test was positif | x |  |  |  |  |
| Adherence assessment (self-reported) |  |  | x |  | x |
| Condom use (self-reported) ^†^ | x | x | x | x | x |
| Sensitization on oral PrEP adherence | x | x | x | x | x |
| Provision of condoms and lubricating gel | x | x | x | x | x |
| Provision of TDF/FTC | x | x | x | x | x |
| Vaccine for HBV | x |  | x |  |  |
| Field visits (peer-educators) |  | x |  | x |  |

Notes: ^†^ Measures done at the study clinic at enrollment, month-6 and month-12 are presented in the paper; ^‡^ At the clinic for enrollment, months 6 and 12, and self-testing in the field for months 3 and 9 ; ^§^ Rapid immuno-chromatographic test for HBV surface antigen and HCV antibodies, Enzyme immunoassays for HBV core and surface antibodies. HBsAg-positivity was used as a surrogate of active hepatitis B infection while HBsAg or anti-HBc-positivity without anti-HBs-positivity defined lifetime hepatitis B infection; CT, *Chlamydia trachomatis*; FTC, Emtricitabine 200 mg; HBV, Hepatitis B virus; HCV, Hepatitis C virus; HIV, Human immunodeficiency virus; NG, *Neisseria gonorrhoeae*; PCR, Polymerase chain reaction; PrEP, Pre-Exposure Prophylaxis; STI, sexually transmitted infections; TDF, Tenofovir Disoproxil fumarate 300 mg.
